# Hydrogen, a Novel Therapeutic Molecule, Regulates Oxidative Stress, Inflammation, and Apoptosis

**DOI:** 10.3389/fphys.2021.789507

**Published:** 2021-12-20

**Authors:** Yan Tian, Yafang Zhang, Yu Wang, Yunxi Chen, Weiping Fan, Jianjun Zhou, Jing Qiao, Youzhen Wei

**Affiliations:** ^1^Research Center for Translational Medicine, Tongji University Affiliated East Hospital, Shanghai, China; ^2^Department of Pediatrics, Taian City Central Hospital, Taian, China; ^3^Department of Microbiology and Immunology, Shanxi Medical University, Taiyuan, China; ^4^Department of Pediatrics, Tongji University Affiliated East Hospital, Shanghai, China

**Keywords:** molecular hydrogen (H_2_), reactive oxygen species, antioxidant, anti-inflammatory, antiapoptotic

## Abstract

Molecular hydrogen (H_2_) is a colorless and odorless gas. Studies have shown that H_2_ inhalation has the therapeutic effects in many animal studies and clinical trials, and its application is recommended in the novel coronavirus pneumonia treatment guidelines in China recently. H_2_ has a relatively small molecular mass, which helps it quickly spread and penetrate cell membranes to exert a wide range of biological effects. It may play a role in the treatment and prevention of a variety of acute and chronic inflammatory diseases, such as acute pancreatitis, sepsis, respiratory disease, ischemia reperfusion injury diseases, autoimmunity diseases, etc.. H_2_ is primarily administered *via* inhalation, drinking H_2_-rich water, or injection of H_2_ saline. It may participate in the anti-inflammatory and antioxidant activity (mitochondrial energy metabolism), immune system regulation, and cell death (apoptosis, autophagy, and pyroptosis) through annihilating excess reactive oxygen species production and modulating nuclear transcription factor. However, the underlying mechanism of H_2_ has not yet been fully revealed. Owing to its safety and potential efficacy, H_2_ has a promising potential for clinical use against many diseases. This review will demonstrate the role of H_2_ in antioxidative, anti-inflammatory, and antiapoptotic effects and its underlying mechanism, particularly in coronavirus disease-2019 (COVID-19), providing strategies for the medical application of H_2_ for various diseases.

## Introduction

Molecular hydrogen (H_2_) is the lightest and most abundant element in the earth's atmosphere. It was considered as a new type of natural antioxidant with low capacity to react with most biomolecules, which has potential therapeutic benefits. The first application of H_2_ for humans is hydreliox, a breathing gas mixture of H_2_, helium, and oxygen (O_2_), which is used to prevent decompression sickness and nitrogen narcosis during very deep technical diving (Lanphier, 1972). Therapeutic applications of H_2_ were first demonstrated in 1975, showing that hyperbaric H_2_ caused marked regression of tumors in mice with skin squamous carcinoma (Dole et al., 1975). In 2007, a Japanese scholar discovered that inhalation of low concentration H_2_ could significantly inhibit cerebral ischemia–reperfusion (I/R) injury and stroke in rats by buffering oxidative stress (Ohsawa et al., 2007). Since then, the biomedical effects, mainly antioxidative, anti-inflammatory, and antiapoptotic effects of H_2_ have been explored with many cellular, animal, and clinical studies. Despite many inaccuracies, selective free radical and inflammation scavenging ability are still the widely accepted mechanism of H_2_. Our previous study also revealed that preinhalation of H_2_ could protect caerulein-induced acute pancreatitis in mice by inhibiting inflammation and oxidative stress in the early stage (Yin et al., 2021). Clinical trials have demonstrated that H_2_ treatment was safe and effective in patients with asthma and chronic obstructive pulmonary disease (COPD) (Wang S. T. et al., 2020; Zheng Z. G. et al., 2021). Furthermore, therapeutic effects in patients with oxidative stress caused by cardiac arrest and inflammation caused by sports have also been shown (Ostojic et al., 2014; Tamura et al., 2020). Most recently and remarkably, to confront coronavirus disease-2019 (COVID-19) pandemic caused by severe acute respiratory syndrome coronavirus 2 (SARS-CoV-2), starting from the Chinese Clinical Guidance (7th edition) for COVID-19 Pneumonia Diagnosis and Treatment issued by China National Health Commission, the inhalation of O_2_ mixed with H_2_ gas (33.3% O_2_ and 66.6% H_2_) has been recommended due to the significant role of H_2_ in ameliorating lung function decline, emphysema, and inflammation in acute or chronic pulmonary disease (Guan et al., 2020a). The therapeutic effects of H_2_ will be highlighted.

Thus, this review will discusses the new application H_2_ and for COVID-19, apart from the scavenger properties of H_2_ against excess reactive oxygen species (ROS) and abnormal inflammation.

## Molecular Hydrogen

### Introduction of Hydrogen Physiology

Molecular hydrogen is a kind of colorless, odorless, and minimal molecule in nature. H_2_ gas is flammable and will burn in air at a very wide range of concentrations between 4 and 75% by volume. Actually, it could quickly diffuse through the alveoli into the blood and circulate throughout the body during breathing; then, given the small relative molecular weight and lack of polarity, H_2_ can quickly penetrate the cell membrane and disperse to the cytoplasm, nucleus, and other organelles to play the biological role. Moreover, H_2_ passes through the blood–brain barrier although most antioxidant compounds cannot. As a kind of mild gas, no cytotoxicity has been reported on the body due to H_2_ so far. H_2_ also has no direct effect on physiology, such as body temperature, blood pressure, pH, or pO_2_ (Zhou et al., 2019). However, it may play an anti-inflammatory and antioxidative role by directly affecting mitochondrial electron transport and neutralizing oxidative stress to alleviate mitochondrial damage, or by balancing intracellular environmental homeostasis and affecting the transcription of key regulatory proteins of inflammation (Ostojic, 2015). H_2_ gas or H_2_-dissolved solution concentration is measurable by gas chromatography. H_2_ concentration also can be specifically detected by an H_2_ electrode. When gas chromatography was used to detect H_2_ content, it was found that there was almost no H_2_ in the arterial blood, venous blood, heart, liver, and other tissues in normal rats (Liu et al., 2014; Chen O. et al., 2017). In mammals, the mammalian cells cannot produce H_2_ because of the deficiency of hydrogenases. However, in human, H_2_-producing bacteria through anaerobic metabolism in the gut produces H_2_ at about 50 to 1,000 mg/day by decomposing lactulose (Hylemon et al., 2018), and very small amounts of H_2_ can quickly escape into the blood circulation and exhale out of the body (8–10 ppm) (Shin, 2014), which can be used to monitor health. Furthermore, lactulose is a safe and tolerable source of H_2_ production (Ito et al., 2012). The inhalation of H_2_ actually increases dissolved H_2_ in arterial blood in a H_2_ concentration-dependent manner, and the H_2_ levels in venous blood were lower than in arterial blood (Ohsawa et al., 2007).

### Routes of Hydrogen Administration

Conventionally, the strategies of H_2_ administration in animal model and human researches are classified into three types, namely inhalation of H_2_ gas, drinking H_2_-dissolved water, and injection of H_2_-dissolved saline. In recent years, nanomaterial delivery systems have also been developed. However, the effects from all delivery strategies are dependent on the solubility of H_2_ in water, saline, or blood, as show in Table 1.

**Table 1:** H_2_ intake route and typical management schemes.

**Intake route**	**Advantages**	**Disadvantages**	**Subjects**	**Action time**	**Action effects**	**Intake protocol**	**References**
Inhalation of H_2_ gas	Ensures the intake time and dose	May be explosive when the concentration is higher than 4%	Rats	120 min	Inhibits cerebral I/R injury; antioxidant	1, 2 or 4% H_2_	Ohsawa et al. (2007)
			Human	7 days	Improves COPD symptoms	66.6% H_2_ 6-8h/d	Zheng Z. G. et al. (2021)
			Rats	4 months	Improves COPD symptoms; anti-inflammation	41.6, 22 or 2% H_2_ once a day for 2 h	Liu et al. (2017)
			Mice	7 days	Improves asthma symptoms; anti-inflammation	42% H_2_ twice a day, 2 h/day	Huang et al. (2019)
			Human	Every day until discharge	Improves COVID-19 severity	33.3% O_2_ and 66.6% H_2_	Guan et al. (2020b)
			Rats	12 h	Alleviates acute pancreatitis; anti-inflammation	2% H_2_	Zhou et al. (2016)
Drinking H_2_-dissolved water	Portable and safe	Limited intake dose	Human	2 weeks	Alleviates sports-related soft tissue injuries	H_2_-rich tablets, 2 g/day	Ostojic et al. (2014)
			Guinea Pigs	10 days	Ameliorates allergic rhinitis; immunoregulation	20 μL/day introduced into nasal passage; 0.6 mmol/L	Xu et al. (2018)
			Human	4 weeks	Reduces inflammation and apoptosis of peripheral blood cells	0.753 mg/L, 1,500ml/day	Sim et al. (2020)
			Mice	10 days	Alleviates EAE symptoms; anti-inflammation	0.36 or 0.89 mM twice a day	Zhao et al. (2016)
			Human	8 weeks	Improves parapsoriasis en plaques	H_2_ water bathing twice a week, 10–15 min ever time	Zhu et al. (2018)
			Rats	60 or 90 min	Relieve retina injury; antiapoptotic	Saturated H_2_ eye drops	Oharazawa et al. (2010)
Injection of H_2_-dissolved saline	Ensures the dose and direct application to the affected area	Invasive and has the risk of cross-infection	Rats	24 h	Alleviates inflammation and apoptosis in myocardial I/R injury	0.6 mmol/L, 10 ml/kg	Yao L. et al. (2019)
			Mice	12 h	Attenuates sepsis-associated encephalopathy; anti-inflammation	0.6 mmol/L, 5 mL/kg	Xie et al. (2020)
			Rats	2 h	Attenuates acute lung injury	2.5 or 10 mL/kg	Zou et al. (2019)
Nanoparticle delivery	Safe and higher H_2_ content per unit volume	Expensive	Rats	3 or 24 h	Attenuates myocardial I/R injury; anti-inflammation and antioxidant	4×10^9^ or 2×10^10^ bubbles	He et al. (2017)

Inhalation of H_2_ gas is the most straightforward therapeutic method and has been widely used since the first report. Inhalation of H_2_ ensures the retention time and dose in the body. Inhaled H_2_ diffuses through the alveoli into the plasma and is transported through the blood to the body. A clinical examination demonstrated that exposure to 2.4% H_2_ gas for 72 h does not affect any physiological parameters, suggesting H_2_ may not have adverse effects (Cole et al., 2021). However, the chemical property of H_2_ is that it burns with O_2_ to form water. It may be explosive and dangerous when the concentration in the air is higher than 4%, but a research reported that H_2_ does not explode if it is <10% when mixed with air or O_2_ (Kurokawa et al., 2019). In addition, reports indicated that the antioxidant effect of H_2_ was dose-dependent, and the concentration of H_2_ in blood and tissues was dependent on the time and concentration of inhalation (Fukuda et al., 2007). In recent years, the administration of a mixture of H_2_ and O_2_ gases (66% H_2_; 33% O_2_), obtained by the electrolysis of water, for research and clinical investigation has become more and more common (Li H. et al., 2017; Chen et al., 2020). The application of high concentrations of H_2_ gas may result in more effective outcomes. The generator represents a safe and convenient alternative to high-pressure gas cylinders and requires no replenishment of supplies.

Drinking water containing H_2_ may be more beneficial since it is a portable, safe, and easily administered way to ingest. H_2_ can be dissolved in water up to 0.8 mM (1.6 mg/L) under atmospheric pressure at room temperature without any change of pH. However, the low solubility of H_2_ in water may not be able to guarantee enough H_2_ concentration in certain local damage models, resulting in its poor bioavailability. Propitiously, drinking H_2_ water can be administered frequently to overcome a short half-life. It was estimated that ~41% of ingested H_2_ *via* H_2_-rich water was retained in the body (Shimouchi et al., 2012). However, the concentration of H_2_ in the brain of rats may be too low when using traditional H_2_ sensors after drinking H_2_ water (Liu et al., 2014). Thus, it is essential to achieve high payload delivery of H_2_ at specific locations to improve the efficacy of H_2_ intervention. In recent years, He et al. (2017) designed a microbubbles (MBs) delivery and monitoring system for H_2_ application, which is a delivery vehicle with which H_2_ gas can be loaded onto the MB shell and can be transported *via* blood circulation. H_2_ content per unit volume of solution was higher when using MBs than using H_2_-saturated saline, implying that H_2_-MBs could be a better method for prevention of myocardial I/R injury in a rat model.

Due to the hydrogen's ability to diffuse through the membrane into the cells, H_2_ bathing research has evolved in therapeutic applications. H_2_ water bathing showed positive effects in the treatment of skin diseases (Zhu et al., 2018; Asada et al., 2019). Expanding the application for H_2_ is preservation of graft organs. Excised grafts were submersed in saturated H_2_-rich water during cold preservation which attenuated cold I/R injury of grafts (Noda et al., 2013), and alleviated chronic graft-vs. -host disease by inhibiting excessive inflammation and oxidative stress (Qian et al., 2021). Moreover, retinal I/R injury was shown in animal models by transient elevation of intraocular pressure and a mechanistic increase in ROS, and this injury was reversed by the continuous delivery of H_2_ saturated eye drops, especially in apoptosis (Oharazawa et al., 2010).

Molecular hydrogen saline injection is a method that can rapidly supply a large amount of H_2_ into the body and allows H_2_ for the direct application to the affected area. However, this method is invasive, difficult for patients to accept, and has the potential risk of crossinfection. In addition, it could be very dangerous if H_2_ is injected directly into the skin or vein. In a study using a rat model, H_2_ was administered orally, injected intraperitoneally or intravenously, or inhaled in its H_2_-water, H_2_-saline, or H_2_ gas forms, respectively. After measuring the concentration of H_2_ by high-quality sensor gas chromatography, H_2_ was present in different concentrations in different tissues (Liu et al., 2014). Thus, H_2_ can reach to most organs or blood independently by the three methods.

However, different administration methods may have different effects. A pharmacokinetics research of a single inhalation of H_2_ gas in pigs showed that H_2_ concentration in the carotid artery peaked immediately after breath holding, and it dropped to 1/40 of the peak value 3 min later. Peak H_2_ concentration in venous blood was much lower than that in arterial blood, which indicated that H_2_ is not simply diffused, but diffuses while being carried by the blood stream (Sano et al., 2020). Hydrogen peaks in inhalation and oral delivery at almost the same time, but the sustaining time of drinking H_2_ water is longer (Sobue et al., 2015). For protein expression, drinking H_2_ water could more significantly downregulate the expression of NF-κB in rats liver tissue than H_2_ inhalation, and the combined effect was even better (Sobue et al., 2015). The concentrations of H_2_ reached a maximum at 5 min after the oral or intraperitoneal H_2_ administration, while intravenous treatment used only 1 min. The decline of the H_2_ concentrations in the blood and tissues observed after reaching the highest level at 5 min was more rapid following intraperitoneal administration than oral administration. The inhalation of H_2_ gas resulted in slower elevation of the H_2_ concentration than that achieved with intraperitoneal, intravenous, or oral administration. However, the elevated H_2_ by inhaling was maintained for at least 60 min (Liu et al., 2014). The fact that inhalation takes a longer time for tissue H_2_ concentration to saturate than ingestion of H_2_ water is counterintuitive, which is because of using especial hermetic tubes filled with pure air to prevent the leakage of H_2_ from the sampling tissue during processing and the application of high-quality sensor gas chromatography in this work. In addition, different methods of H_2_ inhalation give different results, such as masks or nasal tubes. Thus, different administration routes of H_2_ need to be considered according to the needs of the user to guarantee the best acceptable benefits.

## Biological Effects of Hydrogen

### Antioxidant Effect

#### Neutralization of ROS

The disequilibrium between ROS and the antioxidant system causes oxidative stress which is considered as a common initial step for many pathological processes (Burton and Jauniaux, 2011). These ROS include superoxide anion (${\text{O}}_{2}{•}^{-}$), hydroxyl (•OH), peroxyl (RO_2_•), alkoxyl (RO•) radicals, and nitric oxide (NO•), and they primarily come from mitochondrial respiration, NADH/NADPH oxidase, or xanthine oxidoreductase (Dan Dunn et al., 2015). When cell damage occurs, oxidative phosphorylation and electron transport in mitochondria are obstructed, and electrons are leaked to produce excess ROS. On the one hand, the overproduction of ROS causes damage of cell membrane or organelle membrane. Then the lipids are detached from the membrane and further peroxide to generate arachidonic acid and leukotrienes, which contribute to inflammatory pain. Furthermore, ROS produced by neutrophils and macrophages could attack pathogens, which may damage the structure of mitochondria and nucleus of normal cells and subsequently lead to the initiation of apoptosis (Wang et al., 2017). There is no known enzyme specifically to deal with •OH, since the •OH non-selectively reacts instantaneously with the nearest nucleophilic biomolecules. H_2_ is a new type of reductant that can penetrate the cell membrane and neutralize particles that damage the body, •OH and ONOO^−^ in cellular structure, and almost no effects of ${\text{O}}_{2}^{-}$ and H_2_O_2_ which maintains physiological function and internal environment stability (Ohta, 2014). The direct scavenging of the hydroxyl radical according to the chemical reaction of H_2_ + •OH → H_2_O + H• followed by H• + ${\text{O}}_{2}^{-}$ → ${\text{HO}}_{2}^{-}$ was considered as a potential mode of action (LeBaron et al., 2019). As early as 2001, Gharib et al. (2001) found that H_2_ could increase superoxide dismutase (SOD) activity and reduce lipid peroxide malondialdehyde (MDA) level in schistosomiasis-associated liver inflammation model. In 2007, Ohsawa et al. (2007) described its protective benefit against reperfusion oxidative injury *in vitro* and *in vivo*. They demonstrated the protective potential of H_2_ against I/R injury, where H_2_ reduced oxidative stress and scavenged ·OH and ONOO^−^, acting as an electron donor for ROS molecules, but the direct ROS scavenging effect of H_2_ is only confirmed in the acellular experiment. Similarly, after administration with 1.3% H_2_ gas inhalation for 2 weeks in vasculitis mice, the tissue damage was decreased as a result of reduction of ·OH and ONOO^−^ (Kiyoi et al., 2020). By regulating their concentration, they also prevent the production of hydroxyl radicals as they can be converted to •OH radicals *via* the Haber-Weiss and Fenton reaction in the presence of catalytically active metals such as Fe^2+^ and Cu^+^ (Huang, 2016).

Furthermore, the biological and antioxidant effects of H_2_ remain even after H_2_ has been cleared from the body, especially at a low concentration (Dixon et al., 2013), which suggests that the mechanism may have more to do with antioxidant signal modulation than direct free radical scavenging. Nuclear factor erythroid-2 related factor 2 (Nrf2) shifting into the nucleus could lead to the regulation of gene expression involved in defense systems against oxidative stress (Tonelli et al., 2018). Studies have shown that H_2_-rich saline gavage can improve the symptoms of experimental autoimmune encephalomyelitis (EAE) in mice by activating the Nrf2-ARE signaling pathway (Liu et al., 2019). In addition, H_2_ significantly reduces intracellular ROS by upregulating Nrf2 transcription to promote the expression of SOD and glutathione (GSH) and downregulating the expression of NADPH oxidase (Su et al., 2019; Zhao et al., 2019). H_2_ could protect cells against cell death by blocking the abnormal oxidation of phospholipids, reducing the increase in the cell membrane permeability, and thus blocking lipid peroxidation may be another important mechanism of H_2_ antioxidation (Iuchi et al., 2019). Unexpectedly, recent notable studies have suggested that excessive antioxidants increased mortality rates of cancer and cardiovascular diseases (Poljsak et al., 2013; Singh et al., 2018). An ideal antioxidant is expected to mitigate excess oxidative stress, but not to disturb redox homeostasis. H_2_ might be the ideal antioxidant via the rapid diffussion into cells by blood circulation.

#### Regulation of Mitochondria

In addition to focusing on H_2_ neutralizing oxidative stress, the processes upstream of the dysfunction of electron transport chain were focused, which is the first step during mitochondrial oxidative stress. Mitochondria are generally termed the powerhouses of the cell as they produce the 90% of energy in the form of ATP. This process relies on oxidative phosphorylation and accompanies the generation of ROS by forward and reverse electron transfer (Annesley and Fisher, 2019). H_2_ improves mitochondrial dysfunction by preventing the uncontrolled electron leakage from the electron transport chain and is predicted to have the potential ability to regenerate the dysfunction of the cells.

ATP-sensitive K+ channel (mK_ATP_), which is an important energy regulation participant, is located on the mitochondria. For acute myocardial infarction, H_2_ gas could activate mK_ATP_ and regulate mitochondrial membrane potential to equilibrize the level of myocardial NAD+ (the precursor of ATP synthesis) and the production of mitochondrial ATP, thus alleviating myocardial I/R injury (Yoshida et al., 2012).

Coenzyme Q (CoQ) is a key component of the mitochondrial electron transfer chain. The dominant form is CoQ10 in human, while it is CoQ9 in rats. CoQ accepts electrons from Complex I and Complex II and transfers to Complex III, which contributes to the generation of NAD+, the precursor to ATP production and the proton motive force for ATP production (Gutierrez-Mariscal et al., 2020). After H_2_ application, CoQ9 concentrations in plasma and myocardium tissue of rats were significantly increased. In addition, increased CoQ9 improves ATP production *via* mitochondrial oxidative phosphorylation (Gvozdjakova et al., 2020). H_2_ gas has been suggested to enhance the clinical efficacy of nivolumab by increasing CoQ10 of mitochondria to restore exhausted CD8+ T cells (Akagi and Baba, 2020). Therefore, we believe that H_2_ can protect against cell damage by improving mitochondrial function. Improvement of mitochondrial dysfunction is also expected to improve the disordered signal transduction that affects cellular death process, such as Bax and caspase activities (Liu et al., 2016).

Mitophagy plays an important role in maintaining mitochondria homeostasis by eliminating damaged or dysfunctional mitochondria. Fun 14 domain-containing protein 1 (Fundc1) is one of mitophagy receptors localized on the outer membrane of the mitochondrion, which can maintain mitochondrial ATP balance by regulating the mitophagy and interacting with LC3 II. Administration with 2% H_2_ for 3 h promoted Fundc1-induced mitophagy and protected mice from the sepsis-induced liver injury (Yan et al., 2019). In addition, H_2_ exerts neuroprotective effect on oxygen/glucose deprivation neuronal damage in rats, and the increasing expression of mitophagy-related factors, PINK1 and Parkin, indicated that H_2_ is beneficial for ATP generation by promoting mitochondrial autophagy (Wu X. et al., 2018). Animal studies of sepsis have identified the mitochondrial dysfunction may reduce the cellular energy level, resulting in sepsis-related multiple organ failure. For example, in myocardial tissues, H_2_ treatment scavenged ROS by upregulating the heme oxygenase-1 (HO-1, known as heat shock protein 32) to protect sepsis-related multiple organ injury in HO-1/Nrf2 dependent manner (Zhang et al., 2020).

Mitochondrial damage induced by excessive ROS is an important cause of many neurodegenerative diseases. Antioxidant effects of H_2_ intervention on Parkinson's disease or Alzheimer's disease animal models have been shown in previous studies (Wang et al., 2011; Hirayama et al., 2018). However, a randomized double-blind placebo-controlled trial showed that patients with Parkinson's disease who inhaled 6.5% H_2_ gas at 2 L/min for 16 weeks, twice a day for 1 h did not show any beneficial effects even though H_2_ gas was safe (Yoritaka et al., 2021). We believe that this may be related to H_2_ concentration and treatment duration time. Since this clinical trial has only a small number of participants, further study is needed. In conclusion, we assume that H_2_ could equilibrate mitochondrial electron flow, which can explain its ability to scavenge ROS and improve mitochondrial energy metabolism.

### Anti-inflammatory Effect

Inflammation is considered as an adaptive response of the body caused by infection of foreign pathogens or tissue damage, which can cause the aggregation of local neutrophils, monocytes, and other immune cells and release inflammatory cytokines. This process is manifested as lymphocytes and mononuclear phagocytes migrating from veins to the site of injury tissue and getting activated and differentiated into macrophages, in which phagocytes are the main source of growth factors and cytokines (Eming et al., 2017). In addition, excess intracellular ROS can activate inflammatory transcription factors, such as nuclear factor κB (NF-κB), p53, hypoxia-inducible factor-1α (HIF-1α), matrix metalloproteinases, peroxisome proliferator-activated receptor-γ, and nitrosyl radicals, and initiate apoptosis (Mittal et al., 2014; Rimessi et al., 2016; Forrester et al., 2018). Therefore, in the whole pathological process of oxidative stress, inflammation, cell damage, and apoptosis accompany each other and are mutually influenced.

At the early stage of inflammation, H_2_ can reduce the infiltration of neutrophils and macrophages by downregulating the expression of intercellular adhesion molecules and chemokines (Chen et al., 2018), such as early proinflammatory cytokines IL-1β and TNF-α, subsequently decreasing the inflammatory cytokines such as IL-6 and IFN-γ (Zhao et al., 2013). Wang et al. (2015) found that H_2_-rich saline inhibited the activation of crucial inflammatory signaling pathway NF-κB and reduced serum IL-1β, IL-6, and TNF-α levels, thus alleviating the airway inflammatory response caused by a burn in rats. In addition, H_2_ can significantly reduce the expression of NF-κB in liver injury (Tan et al., 2014), hematencephalon (Zhuang et al., 2019), and skeletal muscle injury caused by acute sports (Nogueira et al., 2020), suggesting that molecular hydrogen can affect the inflammatory process by regulating nuclear transcription factors and downstream proinflammatory cytokines. Besides, for the treatment of diseases of inflammation dysfunction, the balance between anti-inflammation and proinflammation should be emphasized. H_2_ also displays an anti-inflammatory effect in I/R cerebral injury or allergic rhinitis animal model by upregulating regulatory T cells (Tregs), which exerts the immunosuppressive function and inhibits the expression of NF-κB (Li et al., 2016; Xu et al., 2018).

Heme oxygenase-1 belongs to the heat-shock protein family, which is a rate-limiting microsomal enzyme involved in heme catabolism. The product, biliverdin, is rapidly reduced to bilirubin, a potent endogenous antioxidant. It could suppress the expression of IL-1β and NF-κB, limiting septic injury (Fujioka et al., 2017). Studies have demonstrated that H_2_ administration increased the HO-1 expression and the number of anti-inflammatory cytokines, IL-10, in human umbilical vein endothelial cells stimulated by LPS and lung tissue of lung-injured mice (Chen et al., 2015). Similarly, we found that preinhalation of H_2_ could prevent acute pancreatitis in mice effectively by enhancing the expression of Hsp60 protein, a heat stress protein, that stimulated synthesis by high temperature to protect itself, in the early stage (Yin et al., 2021). Therefore, we believe that H_2_ can mobilize the body's defense response to play a prominent role in anti-inflammation.

### Regulating Cell Death

#### Apoptosis

Apoptosis is a type of programmed cell death characterized by cell atrophy, apoptotic body formation, and chromatin condensation. The result is clearing cells from the body and minimizing the damage to surrounding tissues, which plays a pivotal role in normal cell turnover and tissue homeostasis. Apoptosis can be induced by both intrinsic and extrinsic signals. The extrinsic apoptotic pathway is initiated by the death receptors on the cell surface, which interact with the tumor necrosis factor receptor and Fas, resulting in the activation of the downstream caspase-8 and subsequent apoptosis. The intrinsic apoptotic pathway is closely related to the antiapoptotic B-cell lymphoma 2 (Bcl-2) and proapoptotic Bax proteins. Both apoptotic pathways converge at a common end-point, leading to caspase-3 activation and DNA fragmentation (Obeng, 2021). H_2_ may exert antiapoptotic effect through scavenging ROS or regulation of gene transcription, which may regulate endogenous apoptosis.

An *in vitro* experiment showed that H_2_-rich medium significantly inhibited ROS formation, maintained cell viability, and inhibited caspase-3 and caspase-9 in intestinal epithelial cells. Moreover, the inordinate expression of Bax and Bcl-2 was also redressed by H_2_ (Qiu et al., 2020). This effect of H_2_-rich water may be achieved through inhibiting the translocation of the apoptotic markers, caspase-3 and Bax, to the mitochondria (Zhang Q. et al., 2018). H_2_-rich water also could play an antiapoptotic role through upregulating the expression of Bcl-2, an important antiapoptotic factor (Mo et al., 2019). Furthermore, H_2_ can activate the MAPK/HO-1 pathway to inhibit neuronal apoptosis and alleviate ischemic brain injury in neonatal mice (Wang et al., 2020) or protect type II alveolar epithelial cells from hyperoxia-induced apoptosis by activating the PI3K/Akt signaling pathway (Wu D. et al., 2018). Interestingly, H_2_ may have different regulatory effects on tumors, that is, it promoted cell apoptosis and inhibited the growth, migration, and invasion of lung cancer and esophageal cancer cells *in vitro* by upregulating the expression of cleaved caspase-3 (Li Q. et al., 2017; Meng et al., 2020), indicating promising application of H_2_ in tumor therapy. Thus, we suggest that H_2_ may play a multiple role, protecting normal cells from damage and inhibiting the proliferation of cancer cells.

#### Autophagy

Autophagy can maintain the energy balance by degrading macromolecular substances, but excessive autophagy will aggravate the inflammation and damage of tissues and organs, such as in sepsis. Autophagy-related proteins play a pivotal role in autophagy detection, including light chain 3 protein (LC3) and Beclin-1. Zhang et al. (2017) illustrated that H_2_ alleviated isoproterenol-induced cardiomyocytes injury through inhibiting autophagy. H_2_ saturated water significantly reduced the expression of autophagy-related proteins LC3 and Beclin-1 in LPS-induced lung injury, suggesting that H_2_ protected tissues from excessive autophagy (Zhang et al., 2015). However, H_2_ could alleviate LPS-induced neuroinflammation by reducing the expression of mTOR in glial cells, increasing the LC3 II/LC3 I ratio, and promoting autophagy (Zhuang et al., 2020). This may be related to different severity of LPS-induced inflammation models. Fundc1, a mitophagy receptor localized at the membrane of the mitochondrion, can maintain mitochondrial ATP balance by regulating mitophagy. Administration with 2% H_2_ for 3 h promoted Fundc1-induced mitophagy and protected mice from the sepsis-induced liver injury (Yan et al., 2019). In addition, studies have shown that the LC3 II/LC3 I ratio and Beclin-1 expression of the damaged cardiomyocytes increased under the regulation of H_2_-rich water, indicating that H_2_ was included in the degradation process of injured mitochondria for intracellular homeostasis (Yao L. et al., 2019). H_2_ can also activate autophagy by inhibiting stress-related p38 and JNK/MAPK pathways (Guan et al., 2019). Similarly, cell apoptosis and autophagy were significantly enhanced in A549 and H1975 lung cancer cell lines treated with different concentrations of H_2_ gas (Liu et al., 2020).

In conclusion, we deemed that H_2_ has a bidirectional regulatory effect on autophagy when autophagy is hyperactivated during inflammation or/and can protect cells and tissues from damage.

#### Pyrolysis

**Figure 1:**
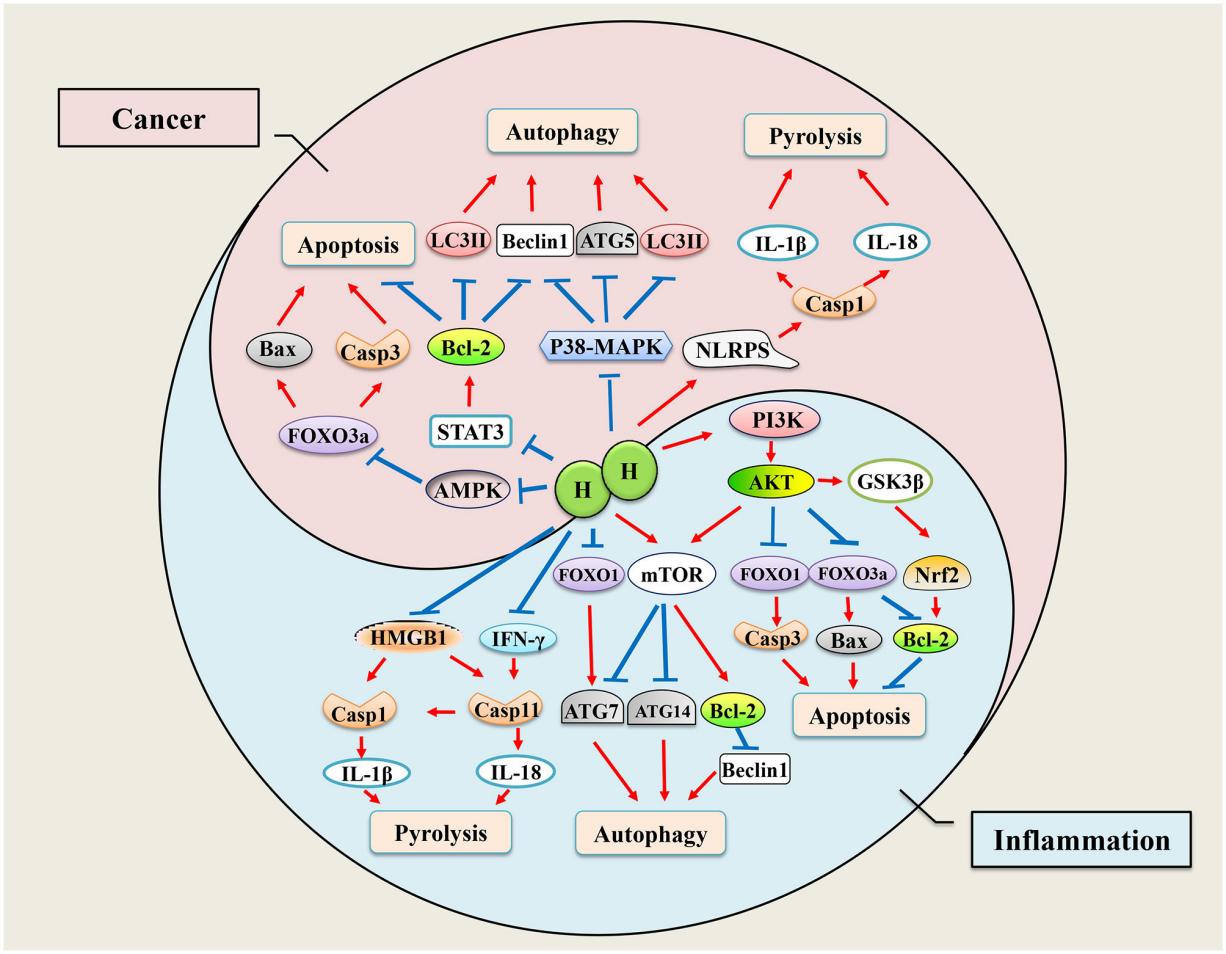
Mechanisms of hydrogen regulates cell death. Hydrogen has bidirectional regulatory effects on apoptosis, autophagy, and pyrolysis. When inflammation occurs, cells initiate apoptosis, autophagy, and pyroptosis to adapt to environmental changes, while hydrogen therapy ameliorates inflammation-induced excessive cell programmed death by regulating transcription of genes. When tumor occurs, hydrogen plays an antitumor role by promoting cell apoptosis, autophagy, and pyrolysis.

Pyrolysis is a programmed death pathway of proinflammatory cells that protects monocytes, macrophages, and other invading pathogens (Man et al., 2017). Although pyrolysis is usually beneficial to the host, excessive pyrolysis can lead to sepsis and septic shock. Caspase-1 is a vital factor in the activation of pyrolysis, and cytokines, IL-1β and IL-18, are the main downstream inflammatory factors in the pyrolysis pathway. The protective effects of H_2_ in septic mice has been demonstrated (Zhai et al., 2015; Xie et al., 2020, 2021). In early subarachnoid hemorrhage brain injury models, H_2_-rich saline can markedly reduce the expression of caspase-1 and inhibit inflammatory response (Shao et al., 2016). Furthermore, in sepsis-related organ injury models, H_2_ treatment significantly reduced the expression of caspase-1 in the damaged organ and the levels of IL-1β and IL-18 cytokines (Yan et al., 2019; Xie et al., 2020). We have known that lung inflation with H_2_ is an effective method to protect donor lungs from I/R injury (Meng C. et al., 2016). Zheng P. et al. (2021) proved that the pyroptosis-related proteins, NLRP3, caspase-1, and the N-terminal of gasdermin D (GSDMD-N), were reduced after lung inflation with 3% H_2_, which means H_2_ alleviated lung I/R injury by inhibiting pyroptosis in Wistar rats. However, H_2_ may play a different regulatory role in tumors. Yang et al. (2020) showed that H_2_-rich water inhibited the proliferation of endometrial cancer cells by triggering the NLRP3 inflammasome/caspase-1 mediated classical pyroptosis pathway and activated the downstream proinflammatory cytokine IL-1β. Endotoxin transporter protein HMGB1 is necessary for activation of caspase-11 pyroptosis pathway (Deng et al., 2018), and the negative regulation of H_2_ on HMGB1 may also play a role in cell pyrolysis (Yu et al., 2019). H_2_ may exert inhibitory effects on cancer cells through apoptosis, autophagy, and pyrolysis. Although there is no direct evidence to explain the mechanism of H_2_ in cell pyroptosis, it is conceivable that the regulation of some nuclear factors and inflammatory factors by H_2_ will interfere with the progress of pyroptosis. The effects of H_2_ on pyrolysis pathway may inhibit tumor cells or protect normal tissues and cells from damage, which is similar to that of apoptosis, as shown in Figure 1.

### Mechanisms and Perspectives of H_2_ for COVID-19

The severe acute bronchitis, pneumonia, and pulmonary fibrosis of coronavirus disease-2019 (COVID-19) caused by severe acute respiratory syndrome coronavirus 2 (SARS-CoV-2) rapidly disseminated across the world in a short span of time, with nearly 185 million confirmed cases and about four million deaths. Most of the cases with COVID-19 manifest as a respiratory illness, starting with a fever and dry cough, followed by shortness of breath with respiratory failure. About 80% of infected people may recover from the illness without hospitalization, the remainder (20%) progress to pneumonia and severe acute respiratory distress syndrome (ARDS), and about 5% of patients develop severe ARDS (Cascella et al., 2021). Currently, few therapies have been proven to be able to rapidly ameliorate the respiratory symptoms and control the disease progression.

When infection occurs, alveolar macrophages and infiltrated immune cells are activated to release proinflammatory cytokines within alveoli and bronchioles. Alveolar hypoxia further induces inflammatory cascades, leading to the production of excess ROS and activation of hypoxia-inducible factors (HIF-1α), and nuclear factor-kappa B (NF-κB) (Dukhinova et al., 2021). Thus, anti-inflammatory and antioxidant therapy are considered essential for severe COVID-19. Inflammatory cytokine storms, caused by an overactive host immune system, can lead to acute inflammatory lung injury and even death (Xu et al., 2020).

Within COVID-19 patients, serum IL-6 and IL-10 levels are positively correlated with disease severity, indicating inflammatory cytokines might be an indicator for disease prognosis (Han et al., 2020). Inhalation with 2% H_2_ significantly reduced the number of inflammatory cells and gene levels of TNF-α, IL-6, IL-17, and IL-23 in the bronchoalveolar lavage fluid in animal model (Liu et al., 2017). Wang S. T. et al. (2020) also showed that 45 min of H_2_ gas inhalation attenuated airway inflammation in asthma and COPD patients by inhibiting the levels of MCP-1, IL-4, and IL-6. Therefore, the early use of H_2_ in COVID-19 patients could potentially suppress the cytokine storms and acute lung injury.

Mitochondrial ROS production usually occurs in the early stage of cell injury, which can lead to destruction of the cell membrane of the alveolar epithelial cells and inactivation of surfactant, thus increasing membrane permeability, leading to increased leakage of proteins into the alveoli during lung injury (Alwazeer et al., 2021). Yet despite high-speed oxygen ventilation, inflammation of the respiratory tract and exudation of viscous mucus in bronchioles and alveoli make oxygenation of blood inefficient in severe COVID-19 as O_2_ cannot easily penetrate mucus plugs. Due to its small molecular weight, H_2_ could elevate forced vital capacity and decrease total respiratory system resistance (Feng et al., 2019). Moreover, H_2_ gas may alleviate dyspnea of patients with COPD by inhibiting bronchiole mucus accumulation and goblet cell hyperplasia (Ning et al., 2013). Moreover, ventilation of highly concentrated oxygen in patients with low SpO2 levels may produce harmful superoxide free radicals, which further paralyzes lung function. Therefore, for COVID-19 patients, inhalation of H_2_ may offer an effective solution to tackle both hypoxia and oxidative stress, thereby reducing downstream cytokine secretion. Different antioxidants were proposed to prevent lipid peroxidation of lung surfactants such as coenzyme Q10 and vitamin E (Rossi et al., 2018; DiPasquale et al., 2020). Studies have shown that, in animal models of airway inflammation, intervention with different concentrations of H_2_ could reduce oxidative stress markers MDA and improve antioxidant enzymes GSH in serum and lung tissue (Zhang N. et al., 2018; Huang et al., 2019). Importantly, it was reported that H_2_-rich medium intervention attenuates irradiation-induced human lung epithelial cell line A549 damage by decreasing the production of ROS (Terasaki et al., 2011). There are more studies showing that H_2_ reduces ROS production in alveolar epithelial cells, attenuates the alveolar epithelial barrier damage, improves alveolar gas exchange, and reduces cell damage (Qiu et al., 2019). Thus, we have reason to believe that H_2_ can be an effective mitigation for COVID-19 pneumonia by neutralizing oxidative stress. In a recent multicenter clinical trial, we noticed that the researchers used a mixture of H_2_ and oxygen (O_2_) gas (66% H_2_; 33% O_2_), generated by electrolyzed water, administered in patients with COVID-19. An improvement in clinical symptoms was seen in a significantly higher percentage of patients in the treatment group, who inhaled a mixture of H_2_ and O_2_, than in patients in the control group, who received classic oxygen therapy, although randomization was not applied because of the urgency to deal with the outbreak (Guan et al., 2020b). Similarly, application of H_2_ improves O_2_ utilization rate, reduces O_2_ consumption, and improves exercise endurance as in healthy humans (Alharbi et al., 2021). Inhalation of the mixed gas of O_2_ and H_2_ can expand the bronchioles and reduce the inspiratory effort, and thus promote the absorption of O_2_ by the alveoli (Zhou et al., 2019). SARS-CoV-2 induced activation of p53 apoptosis signaling pathway in lymphocytes may play an important role in the development of lymphopenia of patients (Xiong et al., 2020). H_2_ may exert antiapoptotic effects in peripheral blood lymphocytes, a benefit to COVID-19 (Sim et al., 2020); moreover, H_2_ can also increase surfactant proteins to further prevent lung injury (Huang et al., 2010), which may be used for prevention and treatment in patients with COVID-19. As a landmark event, recent public reports by China's National Health Commission and the Chinese Center for Disease Control and Prevention, the Chinese Clinical Guidance for COVID-19 Pneumonia Diagnosis and Treatment (7th edition) issued by China National Health Commission recommended the inhalation of O_2_ mixed with H_2_ gas (33.3% O_2_ and 66.6% H_2_), bringing H_2_ to the forefront of contemporary therapeutic medical gas research.

**Figure 2:**
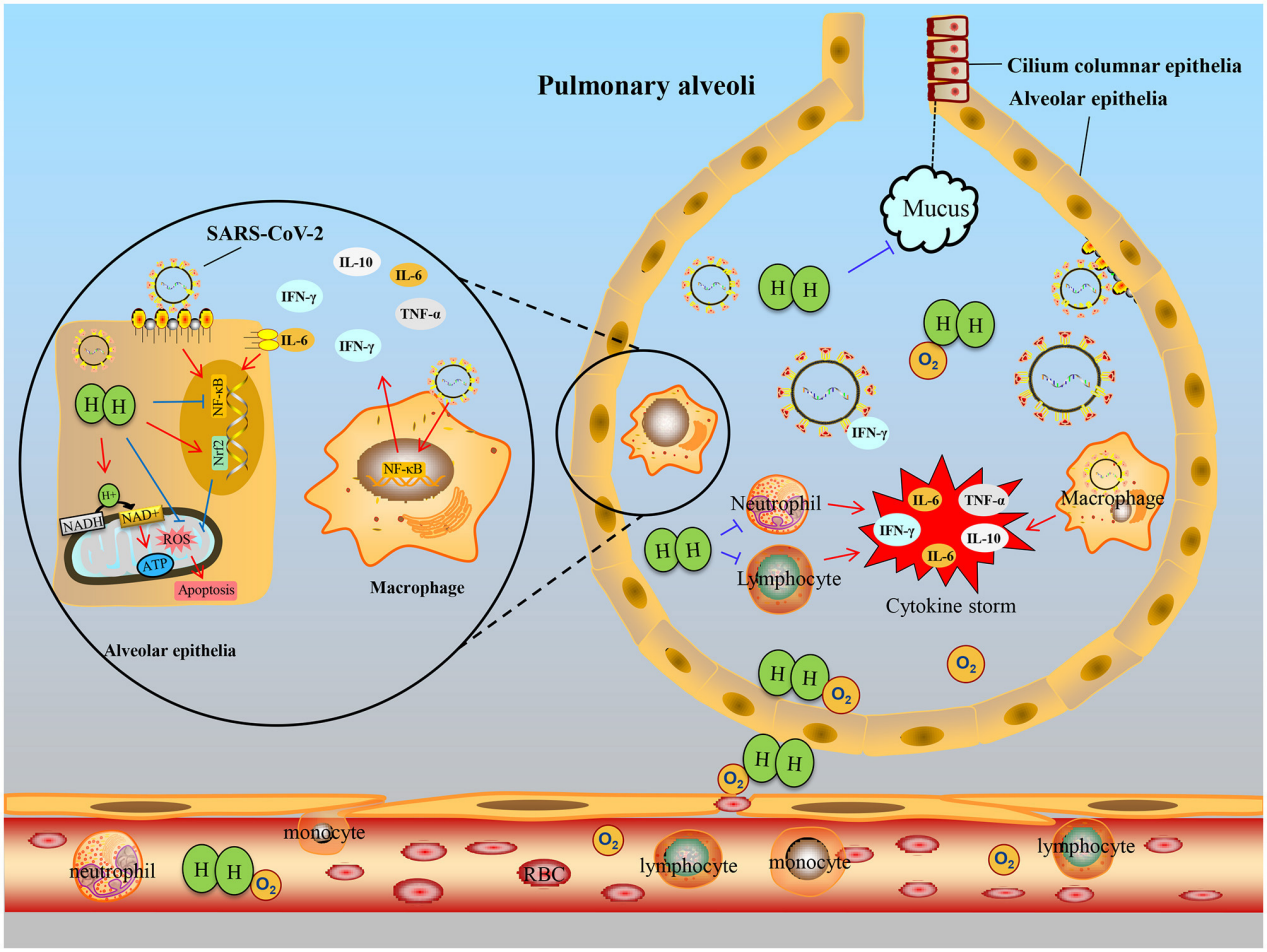
Hypothetical schematic of therapeutic effects of hydrogen for COVID-19. When the SARS-CoV-2 invades the bronchus, the immune defense response is activated, immune cells, such as monocytes and lymphocytes, infiltrate into the alveoli from small vessels, they secrete excessive cytokines IL-6, TNF-α, etc., resulting in cytokine storms and alveolar epithelial cell damage. After hydrogen administration, on the one hand, inflammation can be suppressed by inhibiting NF-κB transcription to reduce activated immune cells, especially macrophages. On the other hand, hydrogen entering alveolar epithelial cells buffers oxidative stress or regulates Nrf2 transcription and inhibits apoptosis. Furthermore, hydrogen can improve dyspnea by inhibiting the secretion of mucus in bronchiole and assisting oxygen diffusion.

Combined with the above researches, we hypothesize that H_2_ inhalation may be a promising approach to alleviate COVID-19 by inhibiting oxidative stress, inflammation, and apoptosis to some extent, as shown in Figure 2.

### Effects on the Immune System

**Figure 3:**
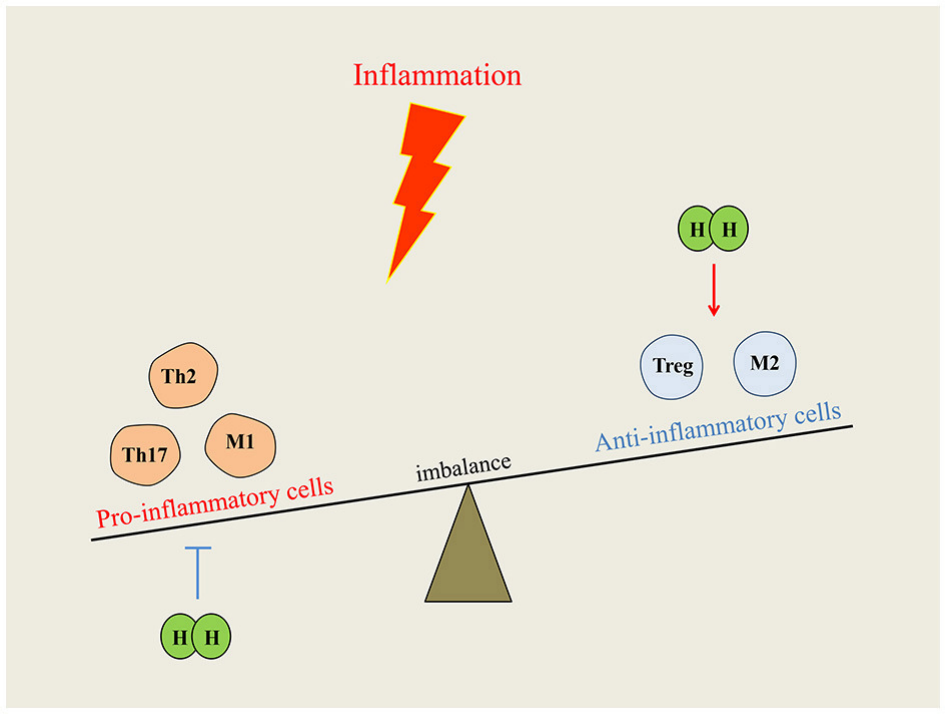
Immunomodulatory function of hydrogen on immune system. When inflammation occurs, the disordered immune cells break the balance of immune homeostasis. Hydrogen intervention could reduce inflammation by downregulating proinflammatory cells or upregulating anti-inflammatory cells, and the imbalance can be redressed.

In many inflammatory diseases, inflammation is mainly caused by the overactivation of cells and proinflammatory substances of the immune system. EAE is the classic animal model for human multiple sclerosis. H_2_-rich water intervention could alleviate EAE symptoms through reducing CD4+ T cell infiltration and inhibiting Th17 cell differentiation in the spinal (Zhao et al., 2016). For immune deficiency, different concentrations of H_2_ can improve the immunodeficiency state and antitumor immune function by increasing the proportion of CD8+ T cells (Akagi and Baba, 2019). Radiation-induced immune dysfunction is the common adverse reaction in many patients subjected to radiotherapy. Studies have shown that pretreatment with H_2_ improved CD4+ and CD8+ T cells and inhibited radiation-induced splenocytes apoptosis, which was against immune dysfunction in mice (Zhao et al., 2014). Moreover, after four-week consumption of H_2_-water, inflammation and apoptosis signaling were significantly downregulated in peripheral blood lymphocytes of healthy adults (Sim et al., 2020). Allergic rhinitis is tissue congestion and edema caused by type I hypersensitivity reaction involved in mast cells and eosinophils activation. It can be relieved with a concentration of 67% H_2_ through inhibiting the inflammatory response of Th2 cells (Huang et al., 2019). In addition, Xu et al. (2018) also demonstrated that H_2_-rich saline ameliorates allergic rhinitis by reversing the imbalance of Th1/Th2. Macrophage polarization and M1/M2 imbalance are pathological features of many inflammatory diseases (Shapouri-Moghaddam et al., 2018). Previous studies have reported that high concentration of H_2_ can improve the IL-4, an anti-inflammatory cytokine, by regulating the M1/M2 balance, and it exhibits significant effectiveness in acute kidney injury (Yao W. et al., 2019), rheumatoid arthritis (Meng J. et al., 2016), and ischemic stroke (Ning et al., 2018). H_2_ was first reported to restore Treg loss in a rat model of chronic pancreatitis, indicating H_2_ also regulates inflammation by mediating Treg. Low dose H_2_ intervention reduced inflammation by promoting Treg proliferation and inhibiting immune overactivation (Chen L. et al., 2017; Xu et al., 2018). Therefore, the different doses of H_2_ may balance immune overactivation or immunodeficiency by regulating immune cells proliferation, as shown in Figure 3.

## Conclusions

Molecular hydrogen seems to exhibit a variety of phenotypes toward improving many pathogenic states by regulating various gene expression. Many studies have shown that H_2_ intervention regulates inflammatory cytokines, scavenges free radicals, and alleviates cell apoptosis injury *in vivo* or *in vitro*, indicating a therapeutic effect of H_2_ on various acute or chronic inflammatory diseases.

**Figure 4:**
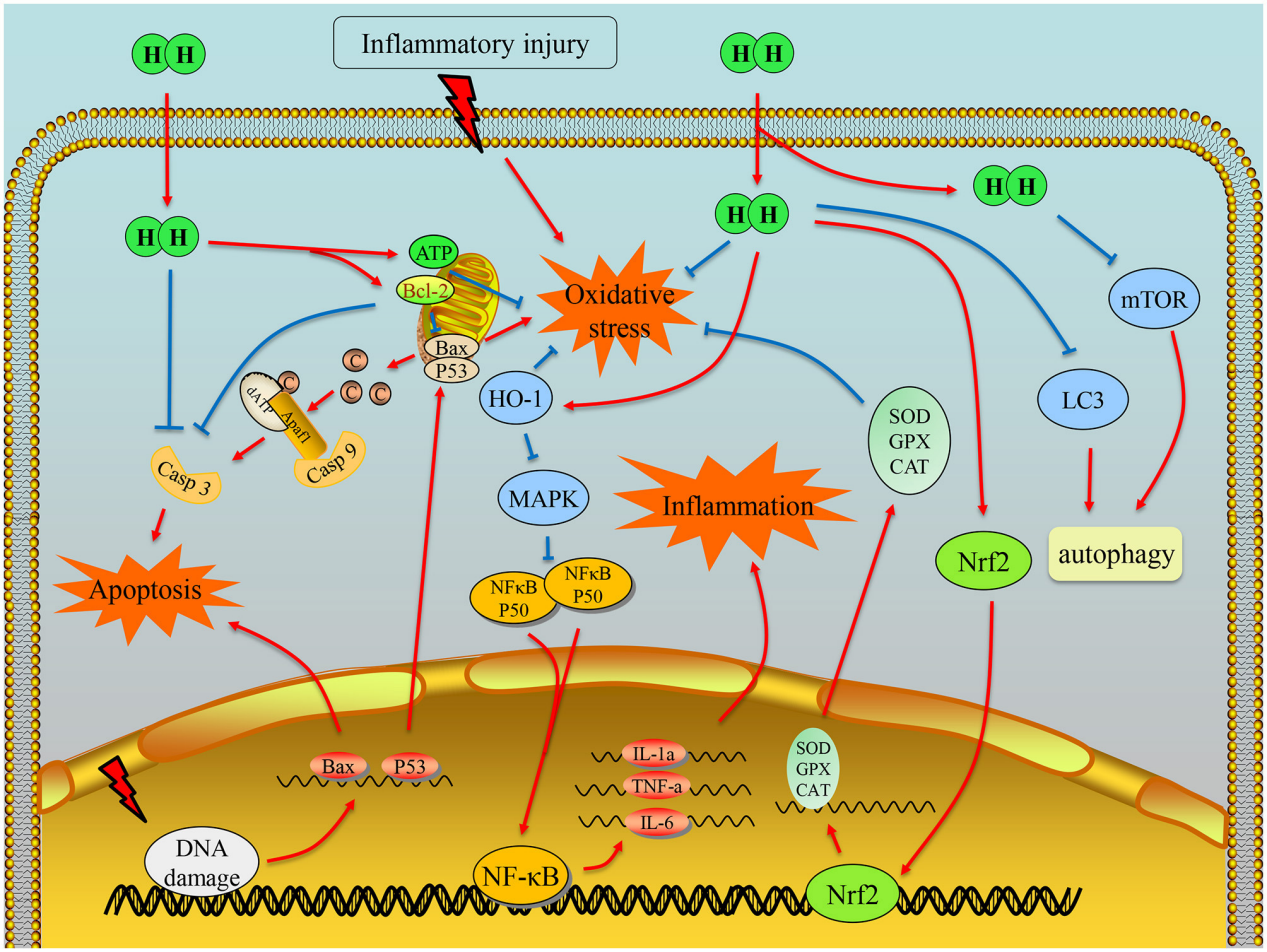
Illustration of the main mechanism of hydrogen therapy in inflammatory diseases. First, hydrogen can scavenge hydroxyl radical due to its chemical property. It can also exert the antioxidation effects by regulating transcription of Nrf2 and energy balance of mitochondria. Moreover, hydrogen could downregulate the transcription of NF-κB, so that the inflammation can be alleviated. With the effects on antioxidation, anti-inflammation, and antiapoptosis factor Bcl-2 or direct interaction with caspase-3, hydrogen can inhibit cell apoptosis.

In conclusion, we consider that H_2_ spreads into cells and regulates the potential imbalance of damaged mitochondrial membrane through electron transfer to reduce the generation of mitochondrial free radicals. It is also involved in the transcriptional regulation of Nrf2 and the occurrence of oxidative stress. For another aspect, H_2_ may inhibit the transcription of inflammation regulatory proteins, NF-κB and Foxp3, in the nucleus, which can further affect the assembly of apoptotic proteins Bax and the phosphorylation of Caspase-3, and eventually prevent the initiation of apoptosis (Figure 4). Moreover, H_2_ acts as a potent antioxidant, and combined with the studies of respiratory system disease, we hypothesize that H_2_ inhalation may provide a meaningful approach to alleviate COVID-19 to some extent.

At present, studies of the prevention and treatment effects of H_2_ on many diseases are still at preliminary stage. Due to this critical difference in the experimental strategy between efficacy testing in animals/cultured cells and clinical trials, further research is needed. It appears that not all studies showed corroborative effects. In order to achieve better results in clinical trials, it is proposed that knowledge involving the effects of excess accumulation, reducing potential, the dosage, the dose duration, and the safety of the antioxidant should all be determined. However, all the findings suggest that H_2_ is a novel and a promising antioxidant agent, and despite its efficacy it has to be further validated and characterized in clinical and animal trials.

## Author Contributions

YW, JQ, and JZ designed and outlined the work. YT, YZ, YW, YC, and WF drafted and revised the manuscript. All authors approved the final version of the article and agreed to be accountable for all aspects of the work.

## Funding

This work was supported by Small and Micro Science and Technology Project grant (No. 20004 to YW) from the National Health Commission.

## Conflict of Interest

The authors declare that the research was conducted in the absence of any commercial or financial relationships that could be construed as a potential conflict of interest.

## Publisher's Note

All claims expressed in this article are solely those of the authors and do not necessarily represent those of their affiliated organizations, or those of the publisher, the editors and the reviewers. Any product that may be evaluated in this article, or claim that may be made by its manufacturer, is not guaranteed or endorsed by the publisher.
